# The influence of sex on pyrazinamide and uric acid serum levels in Brazilian patients treated for pulmonary tuberculosis

**DOI:** 10.1590/S1678-9946202567018

**Published:** 2025-03-17

**Authors:** Carlos Augusto Abreu Albério, Juan Gonzalo Bardalez Rivera, Alberto Camarão de Sousa, Luann Wendel Pereira de Sena, José Luiz Fernandes Vieira

**Affiliations:** 1 Universidade Federal do Pará, Hospital Universitário João de Barros Barreto, Referência em Tuberculose, Belém, Pará, Brazil; 2 Universidade Federal do Pará, Faculdade de Farmácia, Belém, Pará, Brazil; 3 Universidade Federal do Oeste do Pará. Faculdade de Saúde Coletiva, Marabá, Pará, Brazil

**Keywords:** Infectious diseases, Tuberculosis, Uric acid, Pyrazinamide, Arthralgia

## Abstract

Adverse reactions to antituberculosis drugs can lead to treatment abandonment, prolonging the burden of the disease. The role of sex in pyrazinamide exposure and uric acid metabolism raises questions about its influence on the rates of arthralgia and hyperuricemia in patients with tuberculosis. Given the limited evidence in the literature regarding sex-related differences in adverse reaction rates, this study compares serum levels of pyrazinamide and uric acid, as well as the rates of hyperuricemia and arthralgia, between male and female patients with pulmonary tuberculosis. Uric acid levels were measured using the spectrophotometric uricase method, and serum pyrazinamide levels were determined by high-performance liquid chromatography. A total of 88 patients were enrolled in the study. The mean weight, pyrazinamide dosage, and median uric acid levels were similar between sexes. However, the proportion of males with hyperuricemia was higher than that of females. Pyrazinamide maximum concentrations ranged from 10 to 98 µg/mL and were higher in females than in males. The overall rate of arthralgia was 25%, occurring primarily in male patients with hyperuricemia. Serum pyrazinamide levels were higher in patients with arthralgia compared to those without it. No significant correlations were found between drug levels and uric acid in either sex. In conclusion, sex influences pyrazinamide exposure and arthralgia and hyperuricemia rates. Close monitoring of uric acid levels may help improve adherence to tuberculosis therapy.

## INTRODUCTION

Pulmonary tuberculosis (TB) remains a significant public health challenge, with approximately 10.6 million cases reported in 2023^1^. Compliance with treatment is critical for reducing the burden of the disease. However, the frequent adverse reactions (ARs) associated with antituberculosis drugs (Anti-TB) can lead to treatment interruption or abandonment, ultimately affecting treatment outcomes. This, in turn, contributes to the persistence of the disease and the emergence of drug-resistant *Mycobacterium tuberculosis* strains^2,3^.

Pyrazinamide is an important Anti-TB drug due to its sterilizing effect. However, at therapeutic doses, it can cause hepatotoxicity and hyperuricemia^2^. The mechanism behind anti-uricosuric effect of pyrazinamide involves alterations in the activity of enzymes involved in uric acid excretion and metabolism, such as URAT1, OAT2, and xanthine oxidase, leading to decreased uric acid secretion and increased reabsorption. Ethambutol, another Anti-TB drug, is also associated with hyperuricemia, though to a lesser extent, due to its influence on the reduction of uric acid excretion^3,4-6^.

Overall, hyperuricemia does not need the discontinuation of pyrazinamide^4,5^. Although many cases remain asympto­matic, some patients may develop arthralgia, gout, urolithiasis, or nephropathy, conditions that may require drug suspension. Arthralgia is commonly reported in patients undergoing TB treatment and can result from various factors, including hyperuricemia, joint inflammation, extrapulmonary TB, comorbidities, patient immobility, reactive arthritis, or an inflammatory response triggered by the drug via unidentified mechanisms. Additionally, factors like drug dosage, treatment duration, age, and concurrent medications are also implicated in arthralgia in TB patients^2,3,4-6^.

Sex-specific differences in the pharmacokinetics of pyrazinamide may influence the incidence of hyperuricemia-related arthralgia, potentially requiring sex-specific monitoring of adverse reactions to ensure adequate treatment adherence^7,8^. However, this issue has been underexplored in the literature. To address this gap, this study aims to investigate the extent of sex-related differences in pyrazinamide exposure and determine whether these differences contribute to increased rates of hyperuricemia and arthralgia.

## MATERIALS AND METHODS

### Study design, population, and ethics statement

This study was conducted from January to December 2021 at the Basic Health Unit in the Guama district of Belem city, Para State, Brazil. Adults of both sexes with a clinical, laboratory, and radiological diagnosis of newly diagnosed, drug-sensitive pulmonary tuberculosis were included. Exclusion criteria included patients undergoing retreatment or those with resistance to first-line antituberculosis drugs, HIV-positive individuals, and those with diabetes mellitus, liver, gastrointestinal, or kidney diseases, a history of bariatric surgery, prior arthralgia or gout, hyperuricemia, or allergies to antituberculosis medications. The study was reviewed and approved by the Ethics Committee of the Health Sciences Institute at the Federal University of Para (Brazil) under approval Nº 2,911,799. All patients provided written informed consent before participation.

Drug administration and follow-up.

Patients received a daily fixed-dose combination of rifampicin (150 mg), isoniazid (75 mg), pyrazinamide (400 mg), and ethambutol (275 mg). All patients followed the directly observed therapy, short-course (DOTs) strategy and were monitored by the tuberculosis clinical staff at the health unit until treatment completion^9^.

### Blood sampling and laboratory analysis

Approximately 3 mL of blood was drawn from each participant and centrifuged to separate the serum. A portion of the sample was used to assess uric acid levels, while the remainder was stored at −20 °C for subsequent pyrazinamide measurement. Blood samples were collected on the last day of the intensive phase, two hours post-drug administration, corresponding to the maximum concentration of the drug (Cmax)^7,8^.

Uric acid levels were determined using a spectro­photometric technique, using a uric acid assay kit from Sigma-Aldrich^®^, with absorbance readings taken on a UV/VIS spectrophotometer by Thermo Fisher Scientific. The normal reference ranges for uric acid vary by sex and age. In males, the normal range is 3.5 to 7.0 mg/dL, while in premenopausal females it is 2.6 to 6.0 mg/dL. In this study, hyperuricemia was defined as a uric acid concentration exceeding 7.0 mg/dL for both sexes^3^.

Pyrazinamide concentrations were determined using high-performance liquid chromatography (HPLC) with a Flexar system by Perkin Elmer^®^, following liquid-liquid extraction from the serum, with minor modifications^10^. The laboratory-validated procedure demonstrated a detection limit of 2 µg/mL and a quantification limit of 3.3 µg/mL, with linearity ranging from 3.3 µg/mL to 30 µg/mL. Intra- and inter-day variability were 14% and 17%, respectively, with an average recovery rate of 85%.

### Sample effort

A sample size of 33 patients per sex was determined to be the minimum necessary to detect a statistically significant 25% difference in drug concentrations between males and females^7,8^. This calculation aimed to achieve a statistical power of at least 80% and a 95% confidence interval.

### Statistical analysis

Data were reported as frequency of occurrence or as median (range). The Chi-squared (********c2) test or Fisher's exact test was used to compare qualitative variables, while the Mann-Whitney U test was employed to compare quantitative variables between sexes. The Spearman correlation coefficient was used to assess the correlation between serum levels of pyrazinamide and uric acid. A 5% significance level was considered statistically significant.

## RESULTS

A total of 135 patients were undergoing treatment for drug-sensitive TB during the study. However, only 88 patients (65%) met the inclusion criteria and agreed to participate. Males comprised 61% (n=54) of included patients (p=0.028). Table 1 presents the baseline characteristics. Median uric acid levels were similar between sexes, with values of 6.4 mg/dL (range: 4.9-12.0 mg/dL) for females and 6.6 mg/dL (range: 4.9-13.26 mg/dL) for males (p=0.5511). However, the rates of hyperuricemia differed significantly, with 75% of males and 44.4% of females exhibiting uric acid levels above the normal range (p=0.007). The median dose of pyrazinamide administered was 26.2 mg/kg (range: 22.9-29.7 mg/kg) for males and 26.8 mg/kg (range: 23.2-30.8 mg/kg) for females (p=0.2057). The median Cmax of pyrazinamide was significantly higher in females (50.5 µg/mL, range: 10-88 µg/mL) compared to males (42 µg/mL, range: 10-78 µg/mL) (p=0.028). Therapeutic levels of pyrazinamide range from 20 to 50 µg/mL^8^. In 17% of females and 28% of males (p=0.1360), the Cmax was below the therapeutic range, indicating underexposure to the drug. The proportion of positive sputum smears at the completion of the intensive phase was 14.8% in males and 3.7% in females (p=0.0189). Despite this difference, all patients achieved satisfactory outcomes upon completing their treatment.

**Table 1:** Baseline characteristics of patients.

Characteristic	Male (n=54)	Female (n=34)	p-value*
Age, years**	37 (21-48)	33 (19-57)	0.301
Weight, kg**	69 (65-94)	61 (60-79)	0.053
Positive sputum smear at admission, %	80	85	0.697
Fasting blood** glucose, mg/dL	92 (74-101)	84 (69-97)	0.532
Hemoglobin, g/dL**	14 (12-15)	12 (10-13)	0.107
Urea, mg/dl**	26 (17-31)	23 (16-29)	0.593
Creatinine, mg/dL**	0.9 (0.8-1.1)	1.0 (0.7-1.2)	0.787

* p-value set at <0.05%;

** results expressed as median and range.

Arthralgia was diagnosed in 25% of patients (n=22), comprising eight females and 14 males (p=0.0654). However, only five females (22.7%) and eight males (36.3%) with arthralgia had hyperuricemia (p=0.5838). There were no cases of acute gout among the study participants. The median Cmax of pyrazinamide among patients with arthralgia was 45 µg/mL (range: 10-88 µg/mL), which was higher than in those without the condition, who had a median Cmax of 32 µg/mL (range: 14-62 µg/mL) (p=0.021). Additionally, no significant correlation was found between serum uric acid and pyrazinamide levels in males (rs = 0.00026; p=0.7941) or females (rs=0.0338; p=0.5257).

## DISCUSSION

Adequate management of adverse reactions in TB patients is important to ensure satisfactory treatment outcomes^2^. Hyperuricemia and arthralgia rarely lead to treatment suspension as they are generally self-limiting and resolve with the discontinuation of pyrazinamide after the completion of the intensive phase. However, these adverse reactions cause discomfort, and when combined with the improvement in patients’ clinical conditions, they increase the risk of treatment abandonment^2,4-6^.

The baseline characteristics of patients showed a significant prevalence of males, which is a common finding in TB epidemiological studies^5,11^. Despite the significant high weight of males, pyrazinamide doses administered to both sexes were similar and within the range recommended by the Brazilian Health Regulatory Agency^3^. The similarity in doses between males and females is due to the weight-based dose adjustment^9^.

Although the doses received by males and females were similar, the median Cmax of pyrazinamide was significantly higher in females. This finding aligns with studies on the pharmacokinetics and therapeutic monitoring of the drug, which attribute it to the lower clearance and higher oral bioavailability of pyrazinamide in females, which is approximately 26% higher than in males^7,8^. This difference could explain the higher rates of sputum conversion at the completion of the intensive phase, as well as the previously reported lower mycobacterial burden and reduced risk of unfavorable outcomes in females^10^. The higher drug exposure in females also accounts for the lower percentage of underexposure in this group^11^.

The high exposure of females to pyrazinamide could suggest that they would also present elevated uric acid levels. However, male patients exhibited higher uric acid levels than females, which can be attributed to sex-related differences in the metabolism and excretion of the compound, influenced by hormonal factors^12^. These include the suppressive effects of estradiol on xanthine oxidase, as well as differences in testosterone levels and sex hormone-binding globulin levels. Additionally, females have a higher renal clearance rate of uric acid compared to males, which results in a reduced risk of hyperuricemia, estimated to be up to four times lower than that of males^3,12^.

The rate of arthralgia observed in the study aligns with a previous report of 27.7% in patients from Pakistan^13^. However, rates ranging from 16% to 86.3% have been reported in patients receiving pyrazinamide alone or in combination with isoniazid and rifampicin^4-6^. In this study, males exhibited a higher percentage of arthralgia. Hyperuricemia associated with arthralgia was found in approximately 50% of cases, with a similar proportion between sexes. In these patients, signs and symptoms of arthralgia diminished after the completion of the intensive phase of treatment, which corresponds to the withdrawal of pyrazinamide^5^. Thus, arthralgia in TB patients is multifactorial, with hyperuricemia caused by pyrazinamide being one possible cause, as reinforced by the higher Cmax of the drug in patients with arthralgia^2^,4-^6,13^.

There were no significant correlations between pyrazinamide Cmax and uric acid levels in either males or females, reinforcing the role of active metabolites in the anti-uricosuric effect of the drug^2-4^.

The primary limitation of this study was the small sample size of patients exhibiting both arthralgia and hyperuricemia. Nonetheless, there is substantial evidence suggesting that arthralgia during tuberculosis treatment arises from multifactorial causes. Ongoing studies with larger patient populations are being conducted to enhance the robustness of the findings^6,13^.

## CONCLUSION

This study highlights the influence of sex on pyrazinamide uses in patients with tuberculosis, with males exhibiting a higher percentage of hyperuricemia and rates of arthralgia, while females showed higher drug exposure. Sex-specific differences in the rates of adverse reactions during tuberculosis treatment are significant, as they may contribute to treatment abandonment. Therefore, closely monitoring uric acid levels, along with conducting a comprehensive clinical evaluation to identify any prior episodes of gout or arthralgia, is essential for optimizing treatment outcomes and improving patient compliance.
